# Hippocampus discovery First steps

**DOI:** 10.1590/S1980-57642016DN10100011

**Published:** 2016

**Authors:** Eliasz Engelhardt

**Affiliations:** 1 Full Professor (retired), Cognitive and Behavioral Neurology Unit - Institute of Neurology Deolindo Couto (INDC)/Center for Alzheimer Disease (CDA) - Institute of Psychiatry - Federal University of Rio de Janeiro (UFRJ), Rio de Janeiro-RJ, Brazil

**Keywords:** hippocampus, silkworm, discovery, Arantius, Duvernoy, history, hipocampo, bicho-da-seda, descobrimento, Arantius, Duvernoy, história

## Abstract

The first steps of the discovery, and the main discoverers, of the hippocampus are outlined. Arantius was the first to describe a structure he named "hippocampus" or "white silkworm". Despite numerous controversies and alternate designations, the term hippocampus has prevailed until this day as the most widely used term. Duvernoy provided an illustration of the hippocampus and surrounding structures, considered the first by most authors, which appeared more than one and a half century after Arantius' description. Some authors have identified other drawings and texts which they claim predate Duvernoy's depiction, in studies by Vesalius, Varolio, Willis, and Eustachio, albeit unconvincingly. Considering the definition of the hippocampal formation as comprising the hippocampus proper, dentate gyrus and subiculum, Arantius and Duvernoy apparently described the gross anatomy of this complex. The pioneering studies of Arantius and Duvernoy revealed a relatively small hidden formation that would become one of the most valued brain structures.

## RESUMO

Os primeiros passos no descobrimento e os principais descobridores do hipocampo são aqui rastreados. Arantius foi o primeiro a descrever uma estrutura que designou "hipocampo" ou "bicho-da-seda branco". Apesar de muitas controvérsias e denominações alternativas, o termo hipocampo prevaleceu até os dias atuais como o mais amplamente utilizado. Duvernoy apresentou uma ilustração do hipocampo e estruturas próximas, considerada a primeira, de acordo a maioria dos autores, que apareceu mais de um e meio século depois da descrição de Arantius. Alguns autores identificaram outras figuras e textos, que supostamente antedataram os de Duvernoy, nas obras de Vesalius, Varolio, Willis e Eustachio, mas não de modo inequívoco. Considerando a definição da formação hipocampal como compreendendo a hipocampo propriamente dito, giro denteado e subículo, Arantius e Duvernoy aparentemente descreveram a anatomia macroscópica deste complexo. Os estudos pioneiros de Arantius e Duvernoy revelaram uma formação relativamente pequena e ocultada que se tornaria uma das estruturas cerebrais mais valorizadas.

## INTRODUCTION

The hippocampus may be regarded as one of the most studied structures in the brain. Its anatomy was first described over four centuries ago, but its function remained unclear until the beginning of the modern neurosciences era.^1^^,^ 2 Its function (e.g., memory processing) may be affected in various neurological and neuropsychiatric disorders such as Alzheimer's disease, temporal lobe epilepsy, stroke, among others.^1^^-^ 3 Functional and structural imaging of the hippocampus has become an important surrogate marker for defining clinical states.^2^^,^ 3 The structure may be regarded as a complex that comprises, despite lack of consensus, the hippocampus proper, dentate gyrus and subiculum - the hippocampal formation, where many also include subicular related regions and the entorhinal cortex - the hippocampal region, which pertains to the hippocampal system, part of the limbic network.^4^^,^ 5

A brief history is provided tracing the first steps of the discovery, and main discoverers, restricted now to gross anatomical features of the hippocampus, identified at this time as the hippocampal formation as defined above. 

## THE FIRST DESCRIPTION OF THE HIPPOCAMPUS

**Figure 1.**
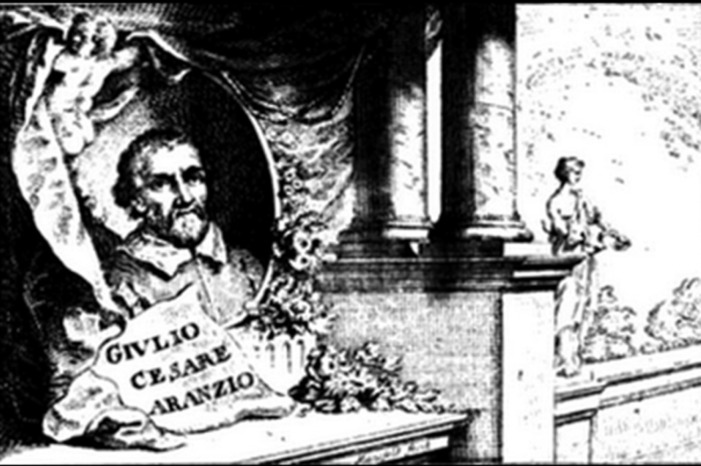
Giulio Cesare Aranzio, from Bologna (illustration from Brambilla, 1781).^8^

The first description and denomination of the structure, practically undisputed, is credited to Giulio Cesare Aranzio (Arantius) (*Julius Caesar Arantius [Bononiensis]*) (c. 1530-1589), an Italian anatomist and surgeon, and pupil of Vesalius^1^^,^ 6 ^-^ 8 (Figure 1). He described and named the anatomical formation in a study of the human brain in the 1^st^ issue of *Anatomicarum Observationum Liber* (Book of Anatomical Observations), which appeared together with the 3^rd^ revised edition of *De Humano Foetu Liber* (Book on the Human Fetus) and the 1^st^ version of *De Tumoribus Secundum Locus Affectus Liber* (Book on Tumors According the Affected Site), published in 1587, compiled together into a single volume.^1^^,^ 2 ^,^ 6 ^,^ 9 The *Anatomicarum Observationum Liber,* concerning the ventricles, choroid plexus and hippocampus, contains five chapters: Chapter I and II describe the ventricles, choroid plexus, and the formation and storage of animal spirits; Chapter III, the main one, provides a description of the hippocampus or silkworm (*vermis bombycinus*) (caterpillar of the *Bombyx mori* moth) and its intraventricular location (inferior or temporal horn) ("ventricle of the hippocampus"); Chapter IV describes the procedure to reach the target structures, while in Chapter V he commented briefly on the ventricles, including the hippocampal one, and the animal spirits produced there. Chapter III was featured in translated and commented form in papers by Lewis'^10^ and Tilney,^11^ whereas Chapters I, III, and IV appeared as selections in the paper of Walther,^12^ presented here as excerpts from the texts in their original and translated forms (Box 1). 

**Box 1.**
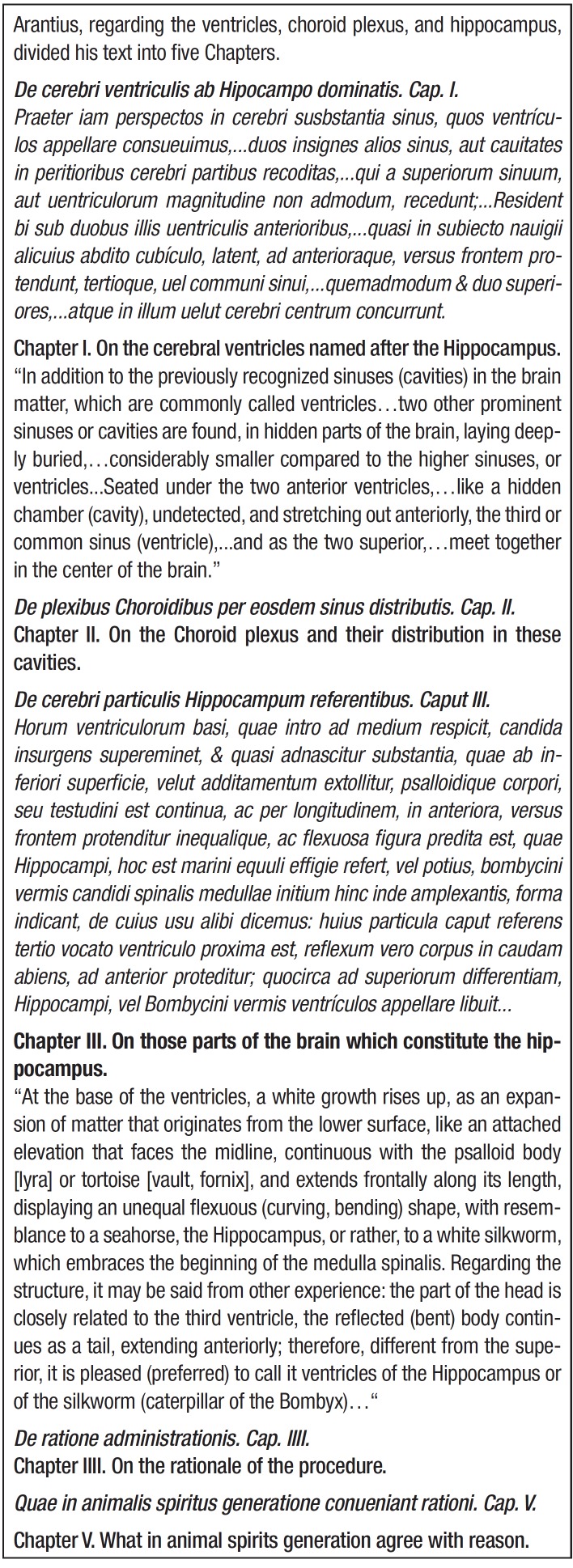
Excerpts from Arantius`' *Anatomicarum Observationum Liber* (Chapters I and III)^9^ (translation checked against those of Lewis, Tilney, and Walther).^10,11,12^

## THE FIRST ILLUSTRATION OF THE HIPPOCAMPUS

According to most authors, the first drawing of the human hippocampus was provided by Johann [Johannes] Georg Duvernoy (*Johannes Georgius Duvernoi*) (1691-1759), a German anatomist and botanist.^13^^,^ 14 He wrote a short essay, *De Sinibus Cerebri* (On sinuses [ventricles, cavities] of the brain), published in 1729 in the *Commentarii Academiae Scientiarum Imperialis Petropolitanae* (Commentaries of the Imperial Academy of Sciences of St. Petersburg), where he presented the text divided into four paragraphs (§1-§4), also denominating the structure as hippocampus or silkworm. It was illustrated with a drawing, Plate XII, of a right human dissected hemisphere, depicting the hippocampus and neighboring structures.^15^

**Figure 2.**
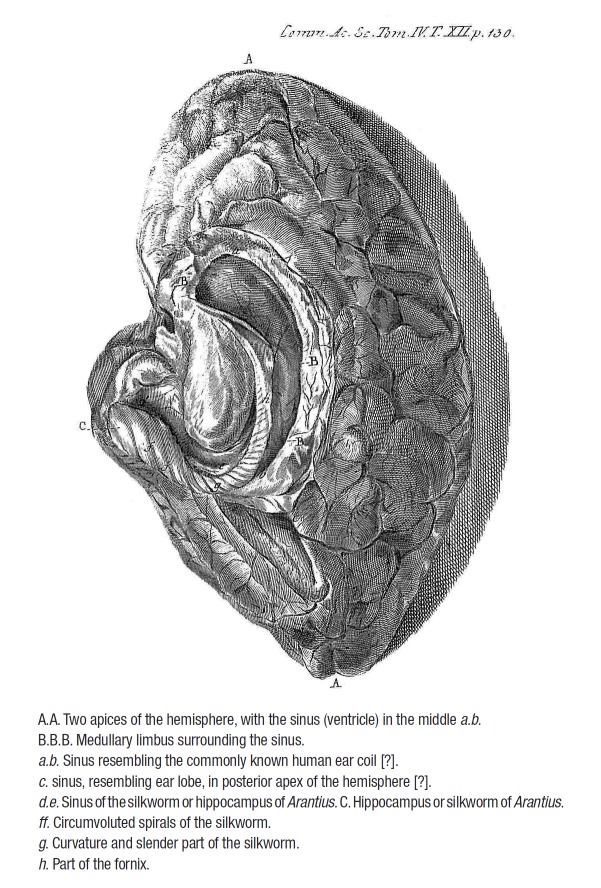
Duvernoy's Plate XII depicting the drawing of a dissected right hemisphere, displaying the hippocampus and neighboring structures.^15^

This material was included as summarized descriptions and some original and translated excerpts (Box 2), as well as a figure with explanations (Figure 2). 

**Box 2.**
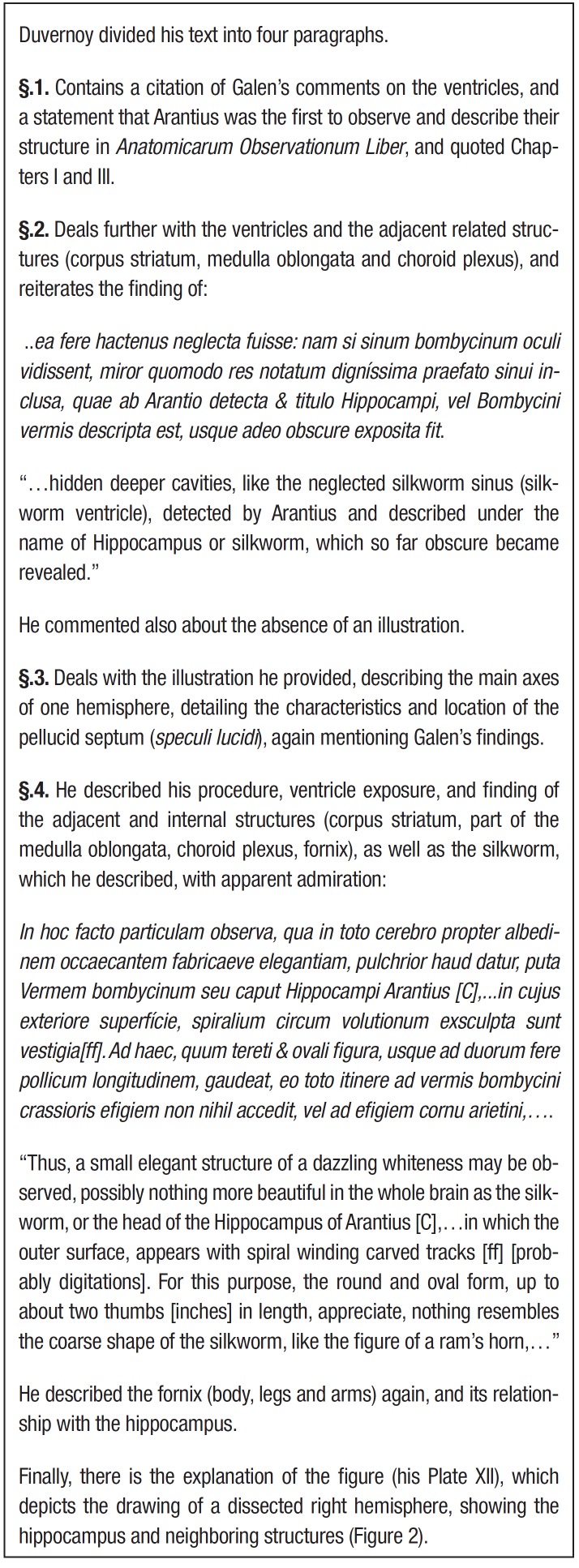
Excerpts from Duvernoy's *De sinibus cerebri* (paragraphs 1-4).^10,15,17^

## COMMENTARIES

The pioneering description of Arantius, and much later Duvernoy's depiction of the hippocampus, revealed a relatively small formation that would become one of the most valued brain structures. It should be noted that up to this point, outstanding anatomical researchers such as Galen, Vesalius, and Willis, had practically overlooked this formation, located deep among numerous other structures in the brain.^10^^,^ 12 ^,^ 16

Arantius was the first to describe this structure, protruding from the floor of the inferior (temporal) horn of the lateral ventricle, which he denominated hippocampus, as to his mind it bore resemblance to a seahorse (or hippocampus, Greek: *hippocampus* [*hippos*=horse*, kampus*=sea monster]) or rather, to a white silkworm (*bombycini vermis candidi*) (white caterpillar of the *Bombyx*)^.1,^ 6 ^,^ 7 ^,^ 9 ^,^ 10 He provided a summarized explanation of the technical procedure, where the dissection was performed with the aid of a bone knife and hands used to reach the deep structures, suggesting that he examined the brain through the exposed lateral ventricle, inspecting the temporal extension, thereby locating the hippocampus with its three parts - head, body, and tail. No illustration of the structure was presented. Thus, his perception of a seahorse or of a silkworm remained rather unclear.^1^ Many controversies arose concerning the description and denomination, as well as alternative designations.^6^^,^ 10 ^,^ 11 However, the term hippocampus has endured until the present day, being the most widely used in the literature.^1^ Other terms emerged designating the structure or its component parts, which will be reviewed on another occasion.^7^^,^ 10 ^,^ 16 ^,^ 17

Duvernoy endorsed Arantius' description of the ventricles and the structures therein, and additionally provided an illustration of the hippocampus and surrounding formations,^15^ regarded as the first by most authors. It must be stressed that it appeared more than one and a half century after Arantius' description. 

However, some authors have identified other illustrations and texts, which they claim predate Duvernoy's depictions listed in chronological order of publication as follows: Andrea Vesalius (*Andreas Vesalius*) (1514-1564) in the 1543 edition of the *De Humani Corporis Fabrica* (On the Fabric of the Human Body), presented drawings and text that might be identified as pertaining to the hippocampus. The structure allegedly illustrated, though not unmistakably, was not described or named.^7^ It should be noted that Vesalius clearly depicted and labeled only the fornix or tortoise, in a horizontally (axially) sectioned brain (e.g. Fifth Figure: *S, T, V. Superior corporis instar fornicis seu testudinis extructi superfícies,...* ("S, T, V. Upper body surface shaped like a fornix [vault] or tortoise..."). *X, X..., corpori testudinem referenti continua.* ("X, X....the reported body of the tortoise continues."),^18^ without any reference to the hippocampus.

Costanzo Varolio (*Constantius Varolius*) (1543-1575), in his book *De nervis opticis* (On the optic nerves), published in 1573, supposedly presented a "rough sketch of the hippocampus", but without any reference to this structure.^10^^,^ 17 The two plates displayed in this book,^19^ as far as can be seen, do not allow this formation to be distinguished. 

Thomas Willis (*Thomae Willis*) (1621-1675), in the *Cerebri Anatome* (Brain Anatomy) of 1664, Chapter X, Figure VII, neglected the structure as he described and depicted a dissected ovine (sheep) brain. However, a formation considered recognizable as the hippocampus was identified,^10^ labeled as *D.D. Corporis callosi margo, qui caudicem medullarem prope Cerebellum amplexabatur.* ("D.D. Margin of the corpus callosum, which embraces the medullary stem close to the Cerebellum."), closely related to *C.C. Fornicis brachia, qui caudicem medullarem e regione glandulae pinealis amplexabantur.* ("C.C. Arms [brachia] of the Fornix, which embraces the medullary stem and the pineal gland.").^20^ The illustration displays a distorted and unclear anatomy of the dissected brain. The recognition of the hippocampus is not convincing. The apparently same dissected brain is presented redrawn in his *Anima Brutorum* (The Soul of Beasts) in the 1672 edition, Plate V, with modifications, and changes in the labels,^10^^,^ 21 where the alleged structure now becomes unrecognizable. 

Bartolomeo Eustachio (*Bartholomeus Eustachius*) (c. 1510-1574) showed in the *Tabulae Anatomicae* (Anatomical Plates), probably commissioned in 1552, but first published by Giovanni Maria Lancisi (1654-1720) only in 1714, almost one and a half century after Eustachio's death, a dissection that displayed a structure presumed to be the hippocampus.^10^ However, this dissection (Plate XVII, Figure V, legend on pp 43-44), depicts the median fornix and the posterior pillars seemingly fusing in an indistinct way and designated by him as the *cornua* (horns), labeled only in the 1717 edition (Plate VI, figure 5... *Insuper fornicem in situ* [...In addition to the fornix *in situ*], *cujusprincipium* [whose body], b. *cornua verò* [the real horns], c, c... (b=body of the fornix, c, c=horns).^22^^,^ 23 There is no mention of the hippocampus. The distinction between the posterior pillars and the horns is not at all clear, as they appear as a single structure, constituting more an illustration of the fornix only. 

Thus, Duvernoy's illustration may be regarded as the first drawing of the structure. If not the first, it may be stated that it was a good depiction^10^ and the best and most representative at the time. 

As originally described, and remains so in the present day, the name "hippocampus" applies to the entire ventricular protrusion. Considering here one of the hippocampal formation definitions, comprising the hippocampus proper, dentate gyrus and subiculum, as described above, Arantius and Duvernoy apparently described the gross anatomy of this complex. Further identification of the component structures occurred later, and will be the focus of another study at a later date.
